# Effect of early intervention for anxiety on sleep outcomes in adolescents: a randomized trial

**DOI:** 10.1007/s00787-021-01795-6

**Published:** 2021-05-07

**Authors:** Bente Storm Mowatt Haugland, Mari Hysing, Asle Hoffart, Åshild Tellefsen Haaland, Jon Fauskanger Bjaastad, Gro Janne Wergeland, Valborg Baste

**Affiliations:** 1 Regional Centre for Child and Youth Mental Health and Child Welfare, Norwegian Research Center, NORCE, Bergen, Norway; 2 Department of Clinical Psychology, Faculty of Psychology, University of Bergen, Årstadveien 17, 5018 Bergen, Norway; 3 Department of Psychosocial Science, Faculty of Psychology, University of Bergen, Bergen, Norway; 4 Research Institute, Modum Bad Psychiatric Hospital, Vikersund, Norway; 5 Department of Psychology, University of Oslo, Oslo, Norway; 6 Sorlandet Hospital HF, Kristiansand, Norway; 7 Division of Psychiatry, Stavanger University Hospital, Stavanger, Norway; 8 Department of Child and Adolescent Psychiatry, Division of Psychiatry, Haukeland University Hospital, Bergen, Norway; 9 Department of Clinical Medicine, Faculty of Medicine, University of Bergen, Bergen, Norway

**Keywords:** Anxiety in adolescents, Insomnia, Sleep onset latency, Sleep duration, Cognitive behavioral therapy, Early intervention

## Abstract

The potential effect of early intervention for anxiety on sleep outcomes was examined in a sample of adolescents with anxiety (*N* = 313, mean 14.0 years, *SD* = 0.84, 84% girls, 95.7% Norwegians). Participants were randomized to one of three conditions: a brief or a standard-length cognitive-behavioral group-intervention (GCBT), or a waitlist control-group (WL). Interventions were delivered at schools, during school hours. Adolescents with elevated anxiety were recruited by school health services. Questionnaires on self-reported anxiety symptoms, depressive symptoms, and sleep characteristics were administered at pre- and post-intervention, post-waitlist, and at 1-year follow-up. Adolescents reported reduced insomnia (odds ratio (OR) = 0.42, *p *< 0.001) and shorter sleep onset latency (*d* = 0.27, *p* <  0.001) from pre- to post-intervention. For insomnia, this effect was maintained at 1-year follow-up (OR = 0.54, *p* = 0.020). However, no effect of GCBT on sleep outcomes was found when comparing GCBT and WL. Also, no difference was found in sleep outcomes between brief and standard-length interventions. Adolescents defined as responders (i.e., having improved much or very much on anxiety after GCBT), did not differ from non-responders regarding sleep outcomes. Thus, anxiety-focused CBT, delivered in groups, showed no effect on sleep outcomes. Strategies specifically targeting sleep problems in adolescents should be included in GCBT when delivered as early intervention for adolescents with elevated anxiety.

*Trial registry* Clinical trial registration: School Based Low-intensity Cognitive Behavioral Intervention for Anxious Youth (LIST); http://clinicalrials.gov/; NCT02279251, Date: 11.31. 2014

**Supplementary Information:**

The online version contains supplementary material available at 10.1007/s00787-021-01795-6.

## Introduction

Anxiety disorders are among the most prevalent mental health problems in adolescence [36]. Adolescence is also characterized by changes in sleep, including reduced sleep duration and increased rates of insomnia [9, 15]. Sleep is associated with anxiety across subtypes of anxiety, with children and adolescents with anxiety characterized by higher rates of insomnia, longer sleep onset latency (SOL), and shorter sleep duration compared to their peers [2, 14, 20, 24, 25, 39, 49]. A bidirectional relationship between sleep and anxiety is reported in longitudinal studies, indicating that poor sleep is a risk factor for anxiety and anxiety a precursor to sleep problems [2, 25, 28]. Inadequate sleep is furthermore assumed to contribute to the maintenance of anxiety, probably due to the negative effects of sleep loss on emotion regulation and cognitive functioning [31, 34].

Treatment studies suggest that sleep problems may moderate outcomes of anxiety-focused cognitive-behavioral therapy (CBT). Less favorable outcomes have been reported for youth with anxiety and co-occurring sleep problems [23, 47], perhaps due to limited capacity to actively engage in anxiety treatment among youth with co-occurring sleep problems. Together, this indicates that sleep problems are highly relevant for the assessment, conceptualization, and treatment of adolescents with elevated anxiety.

The overlap between components in CBT for anxiety and sleep interventions (e.g., relaxation, cognitive restructuring, contingency management, psychoeducation) has raised questions concerning whether anxiety-focused CBT has secondary effects on sleep problems. One could argue that improvements are expected in sleep outcomes when the physical and cognitive arousal associated with anxiety is reduced. Thus, when treating youth with anxiety, improvements in sleep could occur, without targeting sleep directly or applying specific sleep management techniques.

Potential secondary effects of anxiety-focused CBT on sleep have been studied primarily in adult samples. A meta-analysis comprising 19 studies with adults reported moderate effects of CBT for anxiety on sleep problems, across sleep variables and anxiety disorders (i.e., post-traumatic stress disorder, panic disorder with or without agoraphobia, and generalized anxiety disorder) [4]. However, as only a few trials published on anxiety include sleep data, the authors refrained from making firm conclusions about the effect of anxiety treatment on sleep, pending further studies.

A limited number of studies has examined the potential effect of anxiety-focused CBT on sleep in youth

(i.e., children and adolescents) [8, 10, 12, 23, 26, 32, 35, 43]. These studies suggest some effects on sleep outcomes in youth, across different anxiety disorders (see Supplementary Table S1). However, little attention has been given to possible differences in effects depending on the developmental level of the participants. Whereas the frequency of sleep problems may be similar in childhood and adolescence [1, 12], the pattern of sleep problems varies across development. While separation-related sleep problems (e.g., worry about sleeping alone or away from home) are frequent among children with anxiety, dysregulated sleep (e.g., trouble sleeping, sleeplessness, feeling tired or sleepy) is more common among adolescents [8]. This is in line with the more general developmental pattern of sleep problems during adolescence, such as a delay in circadian rhythm, shorter sleep duration, and an increase in insomnia symptoms [9, 40]. Previous studies have focused on a range of sleep-related problems (e.g., nightmares, difficulties falling or staying asleep, parasomnias, refusal to sleep alone, and bedtime resistance) [5, 12, 23, 43]. However, the sleep issues included in the treatment studies are often focused on separation-related sleep problems, leaving us less informed about the effects of anxiety-focused CBT on sleep problems in adolescents. Furthermore, even though previous studies report a reduction in sleep problems in samples comprising both children and adolescents, differences in outcomes between children and adolescents after anxiety-focused CBT are rarely analyzed. Only one previous study has focused specifically on changes in sleep among adolescents with anxiety during CBT. This study [12] includes a sample of youth (*N* = 134, 7–18 years) with anxiety disorders, receiving online individual anxiety-focused CBT. Effects were found for parent-reported sleep outcomes in children in the intervention group compared to the waitlist (WL), whereas regarding adolescents no differences in sleep outcomes were found between the intervention group and the WL. Discrepant findings between age groups could indicate that anxiety strategies in CBT reduce sleep problems more common in childhood (e.g., bedtime resistance, nighttime fears), but target to a lesser degree the sleep problems commonly found among adolescents (e.g., short sleep duration and insomnia). As few studies have focused on adolescents with anxiety and sleep problems relevant for this developmental stage, further studies examining the effects of anxiety-focused CBT for adolescents with anxiety are warranted.

Although laudable for addressing an important clinical question, many of the previous studies have important methodological limitations, such as small sample sizes (*N *< 50) [10, 12, 26, 35, 43], and lack control group [10, 23, 26, 32, 35, 43]. Furthermore, most studies have limited follow-up assessments [8, 10, 23, 26, 35, 43]. Together this impedes our ability to conclude that changes observed in sleep outcomes during anxiety-focused CBT are caused by the treatment. Some studies have included wide age-range samples [8, 10, 23, 35, 43]. This is a problem when examining sleep, where patterns of normality are age-related, with associations between internalizing problems and sleep differing from preschool years to adolescence [16]. Also, several studies have assessed sleep through composite scores, with items drawn from child behavior checklists or anxiety symptom questionnaires [8, 12, 23, 43]. Some of the items assessing sleep are closely related to anxiety (e.g., resisting going to bed and refusal to sleep alone). This represents a problem of item overlap and may inflate the effects concerning anxiety-focused CBT on sleep. Finally, some studies have assessed sleep through parent-report only [12, 23], which is a limitation, particularly in studies including adolescents where both sleep and anxiety may not be easily observable for caregivers.

Most previous studies have examined the secondary effects of anxiety treatment on sleep in youth treated within child and adolescent mental health services (CAMHS) or university clinics. It has been suggested that positive sleep outcomes primarily occur when treatment is initiated during early phases of an anxiety disorder [45]. This points to the relevance of studying sleep outcomes in early interventions with adolescents with elevated anxiety symptoms. To the best of our knowledge, this has been done in only one previous study. The Clementi et al. study [10] reported improvements in parent-rated sleep problems among youth with anxiety symptoms/disorders in an early intervention study. They reported large within-group pre- to post effect sizes, but with a large proportion of the youth (45%) scoring above the clinical cut-off for sleep problems at post-intervention. The study had no control group and small sample size (*N* = 25) within a wide age range (7–16 years). Thus, further studies on early intervention are needed, examining the potential effects of anxiety-focused CBT on sleep. Early interventions aim to reach youth at an early stage of a disorder [13]. Administering interventions in the everyday context of adolescents (e.g., at schools) is expected to increase access to evidence-based interventions and to reduce barriers against seeking help for mental health problems among adolescents [17].

In the current study, we examined two CBT interventions, one comprising 10 sessions (standard-length CBT) and one comprising 5 sessions (brief CBT), both with a duration of 10 weeks. Both interventions have demonstrated efficiency in reducing adolescents’ anxiety- and depressive symptoms [19]. Brief CBT has been defined as sessions reduced by at least 50% compared to the standard 8–12 session interventions [50]. Brief CBT may have the advantage of being more acceptable due to limited resources within primary health services. Furthermore, to our knowledge, no previous studies have examined if the intensity (i.e., the number and duration of sessions) of the intervention affects outcomes of anxiety-focused CBT on sleep. Previous studies on the secondary effects of anxiety treatment on sleep comprise 10 to 20 CBT sessions (Supplementary Table S1). An increasing focus on providing evidence-based mental health interventions to larger groups of youth have boosted the development of brief, less costly and easier-to-access CBT interventions [6]. Thus, examining whether brief anxiety-focused CBT has an effect comparable to standard CBT on sleep problems is relevant, particularly within a primary health care context.

Group-CBT (GCBT) is often applied in the treatment of adolescents with anxiety, has the advantage of providing peer-support, and being less expensive to implement. However, as all previous studies on the secondary effects of sleep among youth include individual CBT, we do not know the effect of anxiety-focused GCBT on sleep outcomes.

The current study utilized data from a randomized controlled trial (RCT), in which sleep was included as a secondary outcome in two GCBT interventions (brief and standard-length programs). As previous studies have found larger improvements in sleep for those defined as responders compared to non-responders after anxiety-focused CBT [8, 35], we also investigated this in the current sample. Thus, the following research questions were examined in a sample of adolescents (*N* = 313, age 12–16 years) with an elevated level of anxiety:

1. Do sleep outcomes improve over time (pre-, post-, and 1-year follow-up) for adolescents with anxiety participating in anxiety-focused GCBT interventions?
2. Does GCBT for anxiety have an effect on adolescents’ sleep. i.e., do adolescents in the GCBT intervention group improve more than those allocated to a waitlist control group?
3. Do anxiety-focused standard-length and brief GCBT have different effects on sleep outcomes?
4. Does the potential effect of anxiety-focused GCBT on sleep outcomes differ between responders (i.e., those improving much or very much from anxiety-focused GCBT) compared to non-responders?

The sleep outcomes included in the present study have not been examined previously in adolescents attending GCBT in primary health care services. Therefore, no specific hypothesis regarding sleep outcomes was made. Furthermore, as comparisons between standard-length and brief CBT have not been addressed earlier, no hypotheses were developed regarding different effects on sleep outcomes. Previous research has found larger improvements in sleep for those defined as responders compared to non-responders regarding anxiety [8, 35]. Therefore, we expected adolescents who had much or very much improvement in anxiety during GCBT to report larger improvements regarding sleep outcomes.

## Methods

### Study design

Data for the current study are secondary outcomes from a RCT where adolescents were randomized to 10-week GCBT interventions (brief- or standard- length CBT) or 10-week delayed-access WL. Following WL, participants were randomized to brief- or standard-length CBT. For further details, see study protocol [21]. Results from the primary outcome measures from this trial have been published elsewhere [19].

### Participants

**Fig. 1:**
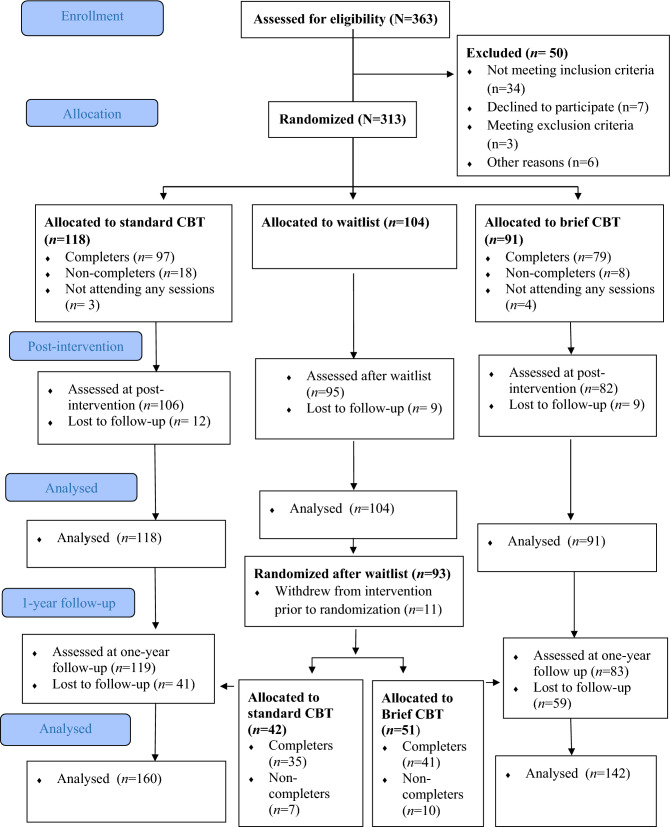
CONSORT Flow diagram reproduced from Haugland et al., 2020

A total of 363 adolescents aged 12–16 years from 18 junior high schools (17 public and 1 private school) were referred for assessment. The schools represented both rural and urban areas. Participants were recruited between October 2014 and November 2016. Inclusion criteria were self-reported *or* parent-reported youth anxiety symptoms (i.e., ≥ 25 on the Spence Children’s Anxiety Scale; SCAS) [42], *and* a minimum level of interference from anxiety in daily life (i.e., a score of  ≥ 1 on the first question on the Child Anxiety Life Interference Scale; CALIS) [30]. Exclusion criteria were (a) problems following group-rules, (b) disruptive behavior, and/or (c) learning problems causing difficulties following a manualized group-program. Whereas fulfillment of inclusion criteria was determined from adolescents’ and caregivers’ scores on SCAS and CALIS, a semi-structured interview developed for the RCT was administered by the providers of the GCBT and gave ground for exclusion. This was a conjoint interview with the adolescent and his/her caregiver(s), assessing the adolescent’s anxiety symptoms, anxious thoughts, avoidance, and his/her goals for the treatment. In addition, adolescents and caregivers were asked whether any of the exclusion criteria might apply. The adolescent’s teacher was consulted to give his/her appraisal of the adolescent, based on observations from the classroom (i.e., the ability to follow group-rules, disruptive behavior, and/or presence of major learning problems). Thus, exclusion was based on information from the adolescents, caregivers, and teachers. A final decision was made in discussion with the principal investigator of the study. Three adolescents were excluded, 34 did not meet inclusion criteria, seven declined to participate, and six were not included as some schools did not manage to recruit enough adolescents to form a group before the semester ended. The final sample comprised 313 adolescents (mean age 14.0 years, *SD* = 0.84, 84.0% girls). See Fig. 1 for the study flow-chart and Table 1 for sample characteristics.

**Table 1:** Baseline demographic and clinical characteristics of participants in group cognitive-behavioral interventions (GCBT) compared to waitlist, and brief GCBT compared to standard-length GCBT

Variable	GCBT versus WL								Brief versus standard GCBT							
	GCBT *n* = 209				WL *n* = 104				Brief GCBT *n* = 142				Standard GCBT *n* = 160			
	Mean	SD	*n*	%	Mean	SD	*n*	%	Mean	SD	*n*	%	Mean	SD	*n*	%
Demographic variables																
Age^a,b^	14.08	0.85			13.81	0.78			13.91	0.86			14.04	0.81		
Sex^c^																
Female			174	83.3			89	85.6			120	84.5			135	84.4
Nationality^c,d^																
Norwegian			200	95.7			101	97.1			136	95.8			156	97.5
Family structure^c^																
Two parents			162	78.8			84	80.8			111	78.2			127	79.9
Single parent			46	22.1			20	19.2			31	21.8			32	20.1
Social class^c,d^																
High			56	26.9			27	26.0			41	28.9			39	20.1
Middle			131	63.0			66	63.5			89	62.7			100	62.0
Low			32	10.1			11	10.6			12	8.5			20	12.6
Internalizing symptoms																
Anxiety symptoms^b^	44.36	16.57			41.56	16.13			42.02	17.20			42.65	16.94		
Depressive symptoms^b^	11.95	6.94			10.54	6.49			11.20	7.02			11.79	7.20		
Sleep outcomes																
Insomnia^c^																
Yes			79	38.0			40	38.5			53	37.6			63	39.4
SOL^c^																
< 15 min			14	6.7			6	5.8			7	4.9			11	6.9
< 30 min			22	10.5			11	10.6			12	8.5			20	12.5
30–59 min			40	19.1			18	17.3			37	26.1			20	12.5
60–119 min			61	29.2			32	30.8			41	28.9			49	30.6
120 + min			72	34.4			37	35.6			45	31.7			60	37.5
Sleep duration wkd^b^																
< 4 h			17	8.4			9	9.0			9	6.5			15	9.8
4–5 h			6	3.0			4	4.0			4	2.9			6	3.9
5–6 h			16	7.9			9	9.0			14	10.1			11	7.2
6–7 h			31	15.3			13	13.0			21	15.1			22	14.4
7–8 h			58	28.7			25	25.0			41	29.5			40	26.1
8–9 h			62	30.7			28	28.0			40	28.8			46	30.1
9–10 h			8	4.0			10	10.0			8	5.8			9	5.9
10–11 h			2	1.0			2	2.0			1	0.7			3	2.0
11–12 h			1	0.5			0	0			1	0.7			0	0
> 12 h			1	0.5			0	0			0	0			1	0.7

*SOL*  sleep onset duration, *wkd*  week day nights

^a^Except for age between GCBT and WL (*p*  <  0.01), no significant differences were found between baseline variables across conditions

^b^Independent-sample *t* test

^c^Pearson *χ*^2^ test

^d^Determined by occupation of the highest-ranking parent, in accordance with the Registrar General Social Class coding scheme and categorized as high, medium, and low

The study was approved by the Regional Committees for Medical and Health Research Ethics in Norway (Approval No 2013/2331).

### Recruitment and randomization

Participants were recruited through multiple formats (e.g., routine student and parent meetings with school nurses, nomination by teachers, and information through media, school, and classroom meetings). Information about the study was also given to those adolescents scoring above mean on a school survey concerning anxiety symptoms. Both self-referral and referral from others were endorsed.

Adolescents and at least one caregiver met with the providers of the GCBT interventions to assess eligibility. Informed written consent/assent was obtained from caregivers and adolescents, followed by baseline assessments and evaluation of inclusion and exclusion criteria.

At each school, sequences of five to eight adolescents were randomly assigned to brief GCBT (*n* = 91), standard-length GCBT (*n* = 118), or WL (*n* = 104). The randomization procedure was determined prior to inclusion and according to a computer-generated random-digit procedure, with groups randomized to all three conditions at each school. A total of 52 intervention-groups were completed (including adolescents re-randomized to brief or standard-length GCBT after WL), comprising 142 adolescents allocated to brief GCBT and 160 adolescents allocated to standard-length GCBT.

A subgroup of adolescents received other treatments for anxiety (i.e., medication, or specialized mental health community services at least once a month) during the interventions (9.9%), WL (6.7%), or the 1-year follow-up (18.5%). A small number received anxiety-medication (SSRIs) pre-treatment (*n* = 4), during the intervention (*n* = 2), or during the 1-year follow-up (*n* = 3).

### Providers and interventions

The GCBT sessions were held at schools, during school hours. Each group was conducted by two providers, comprising mainly school-nurses (*n* = 21), mental health workers from community services (community psychologists *n* = 5, family therapist *n* = 1), or employees from local CAMHS (*n* = 5, e.g., social workers). All providers participated as part of their regular job. The providers were 93.8% women (mean age = 43.2, *SD* = 8.09, range 32–62), most (83.9%) having no prior CBT training. Each provider administered 1–8 groups (mean = 3.3 groups; *SD* = 1.8), with 75.0% administering both interventions.

The standard-length 10 session program (plus two parents-only sessions) was *Cool Kids (CK)*, a CBT program for youth anxiety. The adolescent group-based, school-version was applied [38]. Adolescents attended weekly 90-min sessions. The program comprises workbooks for adolescents and parents.

The brief 5 session program was *Vaag* [37], a group-based CBT program comprising weekly sessions of 45–90 min over four weeks, followed by a final session five weeks later. Session two was a joint youth-parent session.

Both programs include basic CBT-interventions for anxiety, e.g., cognitive restructuring, exposure tasks, and homework. None of the programs cover sleep hygiene or sleep interventions. See study protocol [21] for further details on structure and content of the interventions.

### Training, supervision, and treatment integrity

The GCBT providers received one four-day skills-training workshop focusing on basic CBT-principles for anxiety, programs, and assessment procedures. During the period when the providers administered the interventions, they attended two additional two-day workshops.

Supervision was given by experienced CBT-therapists (*N* = 10) and primarily administered face-to-face. For practical reasons (e.g., geographical distance, weather conditions, a tight time schedule), exceptions could be made, with some supervision sessions delivered digitally or by phone instead of face-to-face. All sessions were videotaped, and these video-recordings were available for the supervisors prior to and during supervision.

Independent raters scored treatment integrity, rating two of the video-taped sessions from each of the 52 groups. Ratings were done by clinical experts, applying the Competence and Adherence Scale for Cognitive Behavioral Therapy (CAS–CBT) [7], covering scorings of adherence to the program (0 = none, 6 = thorough) and competence (0 = poor skills, 6 = excellent skills). Adherence and competence scores for each group (mean of the two rated sessions) ranged from 3.17 to 5.75 (mean = 4.41, *SD* = 0.56) for adherence and 2.75 to 5.88 (mean = 4.18, *SD* = 0.66). Thus, high adherence and good competence were achieved [19].

### Instruments

All measures were administered electronically. Sleep characteristics, and anxiety- and depressive symptoms, were assessed pre- and post-interventions, post-WL, and 1-year after the interventions. Sleep outcomes comprised insomnia, sleep onset latency (SOL), and sleep duration, all previously applied in population-based studies [22, 40]. As sleep outcomes were reported by adolescents only, we applied only adolescents’ self-reported anxiety and depressive symptoms in the current study.

#### Demographic information

Adolescents reported their sex, age, and their own and their caregivers’ country of birth. Social class was determined by occupation of the highest-ranking parent (reported by caregivers and adolescents) according to the Registrar General Social Class coding scheme and categorized as high, medium, and low [27]. Family structure was rated from the question “with whom do you live”, with six possible response alternatives, later categorized as two-parent or single-parent families.

#### Sleep outcomes

*Insomnia* was operationalized according to DSM-5 criteria [22]. The following three criteria were used as an operationalization for insomnia disorder, in line with the DSM-5 criteria: (a) the presence of either difficulty in initiating or maintaining sleep for at least three nights per week; (b) the presence of daytime sleepiness and tiredness for at least three days per week; and (c) duration of the sleep problems for at least three months. A similar definition has been used in other studies, (e.g., [22, 41]). More specifically, insomnia comprised a positive response (“somewhat true” or “certainly true”) to *Difficulties initiating and maintaining sleep* (DIMS) and a positive response (“somewhat true” or “certainly true”) to a joint sleepiness and/or tiredness. Further, insomnia required a DIMS frequency of at least three days per week and a duration of at least three months. The DIMS was rated on a three-point Likert-scale with response options “not true”, “somewhat true” and “certainly true”. Given a positive response (“somewhat true” or “certainly true”), the participants were asked how many days per week they experienced difficulties initiating and maintaining sleep and how long this had been a problem. *Tiredness/sleepiness* was rated by a joint question on a three-point Likert-scale with response options “not true”, “somewhat true” and “certainly true”. If confirmed (“somewhat true” or “certainly true”), adolescents reported the number of days per week they experienced sleepiness and tiredness, respectively.

The adolescents indicated when they usually went to bed at night and their usual rise time in the morning on weekdays and weekends*. Time in bed* (TIB) was calculated by subtracting bedtime from rise-time.

*Sleep onset latency* (SOL) i.e., how long it usually took to fall asleep, was reported in hours and minutes, and further categorized into five levels (from a score of 1 = less than 15 min to 5 = 120 min or more).

*Sleep duration* was calculated separately for weekday and weekend nights and defined as TIB minus SOL. Weekday nights were selected for further analyses. Sleep duration was categorized into ten levels (from a score of 1 = less than 4 h to 10 = 12 h or more).

#### Anxiety and depressive symptoms

*Anxiety symptoms* were assessed by the Spence Children’s Anxiety Scale (SCAS) [33, 42] comprising 44 items, including six positive filler items. SCAS is scored on a 4-point scale, rated from 0 (*never)* to 3 (*always*). SCAS has demonstrated sound psychometric properties [3, 33]. Good to excellent internal consistency was found in the current study, applying Cronbach’s alpha (*α* = 0.91).

#### Responders to GCBT

*Clinical Global Impression–Improvement* (CGI–I) [18], was used to assess change in clinical symptoms. In this study CGI–I focused specifically on the adolescents’ anxiety symptoms and impairment from anxiety. CGI–I ranges from 1 (*very much improved*) to 7 (*very much worse*). The CGI–I was scored by providers of the GCBT, based on a joint parent-youth interview (15–30 min) administered pre- and post-intervention/post-WL. Consistent with previous research [35], adolescents who received a CGI–I score of 1 (very much improved) or 2 (much improved) were considered as responders to GCBT, whereas youth with a CGI–I score ≥ 3 (from minimally improved to very much worse) were defined as non-responders. Three expert scorers, blinded to the original CGI–I scores, rated 20% of the scores based on videotapes of the assessment interviews, with an average agreement between expert scorers and providers of [ICC] (2.1) = 0.81.

### Data analysis

Power calculation for the RCT was performed for the primary outcome measures (i.e., anxiety symptoms), where we aimed to obtain a small to moderate effect size (*d* = 0.40) between the GCBT and WL condition. With an assumed attrition of 10%, a recruitment goal of 323 participants was established [21].

Demographic characteristics, anxiety- and depressive symptoms, and sleep characteristics are presented with mean, standard deviation (SD), numbers and percentages (Table 1). Differences between conditions (GCBT versus WL, and brief versus standard GCBT) pre-intervention were analyzed by *t* tests (continuous variables) and chi-square tests (categorical variables). The same was applied in analyses of potential pre-intervention differences between responders and non-responders.

Logistic mixed-effects models (insomnia) and linear mixed effect models (SOL and sleep duration) (LMMs) were used to analyze possible differences in the change in sleep outcomes between timepoints. To account for dependency within schools and intervention-groups, these were used as random intercepts in addition to individuals in all analyses.

To investigate changes in sleep outcomes across time for all participants (pre- to post-intervention/post WL, and one-year follow-up) a model including time as the fixed effect was provided.

To test differences regarding change in sleep outcomes between GCBT and WL, a model including condition (GCBT/WL), time (pre- to post-interaction / post-WL) and an interaction term between condition and time as fixed effects were provided.

For analyses of differences between brief and standard-length GCBT on sleep outcomes, LMMs were conducted including intervention (brief and standard-length), time and an interaction term between intervention and time as a fixed effect. For the groups re-randomized to GCBT after WL, post-WL scores were used as pre-intervention scores. For insomnia odds ratios (OR) with 95% confidence intervals (CI) are provided, while estimated means, standard error (SE), and p-values for differences between changes in GCBT by time are given for SOL and sleep duration.

Finally, LMMs were used to analyze possible differences between adolescents defined as responders and non-responders to GCBT. Similar models were applied as above, accounting for dependencies within schools and groups as random factors in addition to individuals. Further, responders, time (pre-intervention /or post-WL and post-intervention) and an interaction term between responders and time were included as a fixed effect.

Within- and between-group effect sizes (Cohen’s *d*) were calculated based on estimated means from LMM analyses (unadjusted) and pooled pre-standard deviations. Analyses used the intention-to-treat sample. A significance level of *p* < 0.05 was applied. Missing data were examined by the missing value analysis in SPSS 25 (SPSS/IBM Statistics, Chicago, IL), and handled by a full information maximum likelihood missing data methodology (FIML) in STATA (15.1) (StataCorp, College Station, TX). Missing data originated mainly from participants lost to assessment after WL (*n* = 9; 8.7%), post-intervention (*n* = 21; 10.0%), and at 1-year follow-up (*n* = 100; 33.0%). Nonsignificant Little’s MCAR tests, at pre-intervention (*p* = 0.170), post-intervention (*p* = 0.761), and follow-up (*p* = 0.268) indicated that data on anxiety symptoms, depressive symptoms, insomnia, SOL, and sleep duration were missing completely at random.

## Results

No baseline differences were found between conditions (GCBT versus WL, brief versus standard GCBT) on the demographic variables sex, nationality, family structure and social class, or on anxiety- and depressive symptoms. However, a minor difference in age between GCBT and WL (mean difference 0.27 years, *p* < 0.01) was found. This was considered to have no clinical significance and was not given further attention.

Also, there were no baseline differences on sleep measures between GCBT and WL, or between brief and standard-length GCBT (Table 1). Furthermore, no differences were found between responders and non-responders regarding insomnia, SOL, or sleep duration.

Eleven youth withdrew from the study post-WL, before being re-randomized. Among those randomized to GCBT, 83.4% (*n* = 252) were defined as completers (i.e., attended ≥ 7 sessions of standard-length or ≥ 4 sessions of brief GCBT). No difference in retention was observed between the two interventions (*p* = 0.68). Post hoc comparisons of completers versus non-completers showed no baseline differences regarding participants’ age, sex, social class, family structure, or anxiety- or depressive symptoms. Also, no differences were found regarding any of the sleep outcomes (*p* > 0.09).

### Time effect

Significant time effects were found (pre-, post- and 1-year-follow-up), with better outcomes over time for insomnia and SOL. Changes from pre- to post-intervention were OR = 0.42 (95%CI [0.26 to 0.68], *p* ≤ 0.001) for insomnia and mean changes −0.33 (95%CI [−0.49 to −0.18], *p* < 0.001) for SOL. At 1-year follow-up the effect was maintained for insomnia, with change from pre-intervention to 1-year follow-up OR = 0.54 (95%CI [0.32 to 0.91], *p* = 0.020), but not for SOL where the mean change was −0.16 (95%CI [−0.33 to 0.02], *p* = 0.075).

For sleep duration no change was found between pre- and post-intervention, with mean change:0.10 (95%CI [− 0.10 to 0.310], *p* = 0.30). Shorter sleep duration was observed from pre-intervention to 1-year follow-up with mean change: − 0.31(95%CI [− 0.53 to − 0.09], *p* = 0.006).

**Fig. 2:**
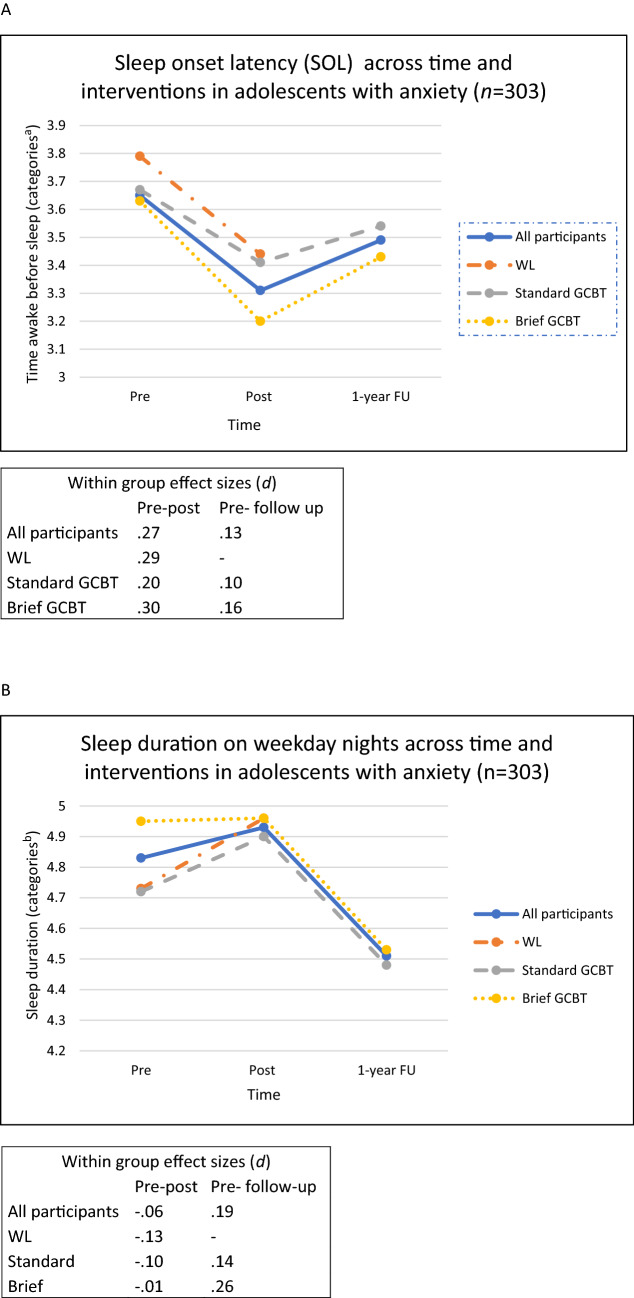
Sleep onset latency and sleep duration across time and interventions for adolescents in early interventions. A: sleep onset latency and B: sleep duration. ^a^1 = less than 15 min, 5 = 120 min or more, ^b^1 = less than 4 h, 10 = 12 h or more

Within-group effect sizes (*d*) for SOL and sleep duration between different time points were small and varied between − 0.06 to 0.27 (Fig. 2).

### GCBT compared to WL

Table 2 presents OR for insomnia, comparing changes in CGBT and WL, whereas Table 3 presents estimated means, within-group effect sizes, and differences in change between GCBT and WL for SOL and sleep duration. Adolescents in the GCBT group did not differ from the WL group from pre- to post-intervention on any of the sleep outcome variables (insomnia *p* = 0.077; SOL *p* = 0.606; sleep duration *p* = 0.738).

**Table 2:** Odds ratio for adolescents' self-reported insomnia, comparing participants in GCBT and WL pre- to post-, and brief and standard length GCBT pre- to post-intervention and at 1-year follow-up

	Intervention				Interaction between time and intervention
	GCBT		WL		
Insomnia	% (*n*)	OR (95% CI)	% (*n*)	OR (95% CI)	*p*
Pre	38.0 (208)	1.00	38.5 (104)	1.00	
Post	25.3 (186)	0.38 (0.21–0.68)	36.8 (95)	0.88 (0.43–1.82)	0.077^a^

	Brief GCBT		Standard GCBT		*p*
	% (*n*)	OR (95% CI)	% (*n*)	OR (95% CI)	
Pre	36.0 (139)	1.00	38.8 (160)	1.00	
Post	23.3 (120)	0.37 (0.18–0.75)	28.6 (133)	0.46 (0.24–0.89)	0.641^b^
Follow-up	29.6 (71)	0.71 (0.32–1.59)	29.3 (116)	0.45 (0.23–0.89)	0.397^b^

Brief GCBT = Vaag, Standard GCBT = Cool Kids

*GCBT* group cognitive behavioral therapy, *WL* waitlist

^a^*p*-value for differences in change between GCBT and WL from pre- to post

^b^*p*-value for differences in change between brief and standard length GCBT from pre- to post-intervention, and from pre-intervention to 1-year follow-up

**Table 3:** Estimated means and effect sizes of adolescents' self-reported sleep onset latency and sleep duration comparing participants in GCBT and WL pre- to post-intervention

Variable	*n*	GCBT	WL	Cohen* d*	Difference btw WL and GCBT		
	GCBT/WL	Mean (SE)	Mean (SE)	GCBT/WL^a^	Mean change	95%Cl	*p*^b^
Sleep onset latency (SOL)^c^							
Pre	209/104	3.74 (0.10)	3.79 (0.13)				
Post	187/95	3.31 (0.10)	3.44 (0.14)	0.35/0.29	− 0.07	− 0.35 to 0.21	0.606
Sleep duration weekdays^d^							
Pre	202/100	4.73 (0.13)	4.73 (0.18)				
Post	187/95	4.90 (0.14)	4.96 (0.18)	− 0.10/− 0.13	− 0.07	− 0.46 to 0.32	0.738

Estimated means from linear mixed models. Differences between intervention and waitlist by mean difference in change in confidence intervals, effect sizes, and *p* values

*GCBT* group cognitive behavioral therapy, *WL* waitlist

^a^Within-group effect size (Cohen *d*) pre- to post- for GCBT and WL

^b^*p*-value for differences in change between GCBT and WL from pre- to post.

^c^1 = less than 15 min to 5 = 120 min or more

^d^1 = less than 4 h to 10 = 12 h or more

### Brief versus standard-length GCBT

No differences were found between brief and standard-length GCBT for insomnia (Table 2), SOL (*p* = 0.600), or sleep duration (*p* = 0.638) (Table 4). Estimated means, within-group effect sizes, and differences in change between brief and standard-length GCBT regarding SOL and sleep duration are reported in Table 4. Small within-group effects were found across time for SOL for both brief and standard GCBT (*d*s ranging from 0.10 to 0.30). For both interventions adolescents reported a small decrease in sleep duration between pre-intervention and 1-year follow-up (*d*_standard GCBT_ = 0.18 and *d*_brief GCBT_ = 0.14) (Table 4 and Fig. 2).

**Table 4:** Estimated means and effect sizes of adolescents' self-reported sleep onset latency and sleep duration comparing brief and standard length GCBT pre- to post-intervention and at 1-year follow-up

Variable	*n*	Brief GCBT	Standard GCBT	Cohen *d*	Difference between Brief and Standard GCBT		
	Brief/Standard	Mean (SE)	Mean (SE)	Brief/standard ^a^	Mean change	95%Cl	*p*^b^
Sleep onset latency (SOL)^c^							
Pre	140/160	3.63 (0.12)	3.67 (0.12)				
Post	121/134	3.20 (0.13)	3.41 (0.12)	0.34/0.20	− 0.16	− 0.48 to 0.15	0.31
Follow-up	72/117	3.43 (0.15)	3.54 (13)	0.16/0.10	− 0.06	− 0.42 to 0.30	0.73
Sleep duration^d^							
Pre	137/153	4.95 (0.16)	4.72 (0.15)				
Post	117/128	4.96 (0.17)	4.90 (0.16)	− 0.01/− 0.11	− 0.17	− 0.56 to 0.23	0.414
Follow-up	71/113	4.53 (0.19)	4.48 (0.17)	0.26/0.14	− 0.18	− 0.63 to 0.27	0.436

Estimated means from linear mixed models. Differences between brief and standard GCBT by mean difference change in confidence intervals, effect sizes, and p values in Brief = Vaag; Standard = Cool Kids; GCBT = group cognitive behavioral therapy

^a^Within-group effect size (Cohen *d*) pre- to post-intervention, and pre-intervention to 1-year follow up

^b^*p*-value for differences in change between brief and standard length GCBT from pre- to post-intervention, and from pre-intervention to 1-year follow-up

^c^scores from 1 = less than 15 min to 5 = 120 min or more

^d^scores from 1 = less than 4 h to 10 = 12 h or more

### Relationship to response from GCBT

No differences were found between those rated as responders (*n* = 134) versus non-responders to GCBT (*n* = 124) on any of the sleep outcomes (insomnia *p* = 0.632; SOL *p* = 0.781; sleep duration *p* = 0.741).

## Discussion

The present study examined whether group-based cognitive-behavioral therapy (GCBT) for anxiety, delivered as early intervention, had secondary effects on sleep problems in adolescents with elevated anxiety. The results demonstrated improvements in sleep for all adolescents (aged 12–16 years), with reduced rates of insomnia and shorter sleep onset latency (SOL) from pre- to post intervention. Furthermore, a decrease in insomnia after GCBT was maintained at 1-year follow-up. Also, the adolescents reported shorter sleep duration from pre-intervention to 1-year follow-up, probably a result of expected age effects [9]. However, no differences in sleep outcomes were found when comparing adolescents receiving GCBT to the waitlist group (WL). Also, no differences in sleep outcomes were found between the two GCBT interventions (i.e., brief and standard-length GCBT). Finally, adolescents defined as responders to GCBT (i.e., those with improved anxiety at post-intervention) did not have larger effects of GCBT on sleep outcomes compared to non-responders.

In the following, we discuss the findings in view of sleep problems in anxious adolescents. The discrepancies in findings compared to previous research will also be discussed considering differences in methods and samples between studies. The findings of no changes in sleep outcomes during anxiety-focused CBT are in line with results reported by Donovan et al. [12]. When comparing anxiety-focused CBT and WL, they found reduced sleep problems in children with anxiety, but no improvements in sleep outcomes among adolescents. Thus, effects on sleep during anxiety-focused CBT may differ between children and adolescents. One reason for the lack of effect of CBT on sleep outcomes in adolescents could be that the anxiety management skills included in CBT affect to a lesser degree those sleep problems more commonly found in adolescents (e.g., circadian rhythm shifts, difficulties initiating sleep, and/or short sleep duration) [9, 20, 39, 49]. The findings from the present study and from the study of Donovan et al., [12] suggest that we need to further examine the effect of anxiety-focused CBT on sleep. It would be of great interest to explore mechanisms that could potentially contribute to improved sleep among adolescents during CBT (e.g., reducing anxious cognitions, decreasing worrying and/or rumination, improving emotion regulation). This could guide us in how to improve current CBT programs and determine what sleep strategies should be included to better address the sleep problems commonly found among anxious adolescents.

Methodological differences must also be taken into consideration when discussing discrepancies between our findings and results from previous studies. As many of the previous studies on sleep outcomes during anxiety-focused CBT do not include control groups, we cannot exclude the possibility that the changes observed are spontaneous improvements and not effects of CBT. Also, in the present study significant pre- to post effects were found for insomnia and SOL across time. However, when comparing GCBT and WL, the changes in sleep outcomes were non-significant. Another RCT (*N* = 488, 7–17 years) reported improved sleep after anxiety-focused CBT compared to a placebo intervention [8]. However, the majority of participants in this study were children (74.2% under 13 years) [46]. To confirm or contest the findings in the present study we need further RCTs focusing on anxious adolescents.

Our sample comprised adolescents in early interventions, whereas most previous studies include clinical samples of youth, treated in CAMHS or university clinics. On average, early intervention studies on youth anxiety demonstrate small effect sizes [44]. Thus, with an even larger sample than included in the present study, one might have achieved significant results. On the other hand, treatment of anxiety has been suggested to positively impact sleep, particularly in the early phases of a disorder [45]. If this is the case, effects on sleep outcomes could be expected from the GCBT interventions in the current study.

Contrary to previous research we examined CBT delivered in groups. GCBT has proved to be equally effective as individual CBT in treating anxiety disorders [48]. In individual treatment, however, therapists might have more room for tailoring the manual to the needs and concerns (e.g., sleep problems) of the individual youth. Hence, we cannot exclude the possibility that there are differences between GCBT and individual CBT regarding the effect on sleep outcomes. As anxiety-focused CBT is often administered as group treatment, it is important to examine further if this is a format less likely to affect co-occurring sleep problems in youth with anxiety.

The lack of difference between brief and standard-length GCBT on sleep outcomes implies that just adding more sessions or time with the therapist does not increase the effect of GCBT on sleep outcomes. If we want to improve the sleep of adolescents in anxiety treatment, we probably need to address the sleep issues directly, rather than merely adding more sessions focused on anxiety-management and expect a spill-over effect to sleep.

In contrast to our findings, two previous studies have demonstrated stronger effects during CBT for anxiety on sleep outcomes in youth defined as responders to the anxiety treatment [8, 35]. Although we applied the same definition of responders/non-responders, no difference in sleep outcomes was observed in the current study. One of the previous studies applied a composite measure with items from anxiety- and symptom checklists as sleep outcome [8], and the other found effects only on parent-reported sleep outcomes [35]. Thus, differences regarding sleep measures and informants are possible explanations for these discrepancies in findings between our study and previous research.

### Strengths and limitations

The adolescents were recruited by school health services, and the interventions were delivered in the everyday context of the adolescents. Furthermore, attrition was low, and a large percentage (84.3%) were defined as completers of the interventions. This indicates a high external validity of the findings, and within some limitations, a possibility of generalizing to adolescents with anxiety in primary health services.

Providers of the interventions were health personnel who administered the interventions as part of their regular job. High levels of adherence and competence were observed. A further strength of the study was the inclusion of measures specifically assessing sleep, rather than sleep-related outcomes that often include overlapping items between sleep and anxiety. Including adolescents’ self-reports rather than parent-reports is also a strength in the current study. Although parents in many instances are considered more reliable reporters than youth, this may not be the case when assessing sleep difficulties (e.g., SOL) and anxiety symptoms. These are issues that are not always easily observable for caregivers. However, multi-informant assessments are important in research on youth mental health [11], and parent-reports could have been a supplement to the youth’s self-reports.

The study has several limitations. It was designed to examine the effectiveness of school-based GCBT for anxiety. Power calculation was not conducted to ensure a sample size to detect secondary effects on sleep outcomes. Furthermore, despite efforts to include both sexes, the sample comprised a large proportion of girls. The sample was demographically restricted, with an overrepresentation of upper to middle class, two-parent families, and adolescents with Norwegian nationality. These characteristics represent a limitation for the generalization of the finding.

Questionnaires were used to assess anxiety. However, including diagnostic interviews to determine the diagnostic status of the adolescents would have strengthened the study. All three sleep variables were categorized. Whereas this provided a more overall and organized presentation of the data, using continuous sleep variables would have given more variance and perhaps larger nuances in the findings.

The exclusive use of subjective measures of sleep is also a limitation. However, a recent study reported high correspondence between subjective and objective measures of sleep duration among adolescents [29], supporting the use of self-reported sleep measures. Furthermore, in the present study sleep duration was based on SOL subtracted from time in bed. To gain a more accurate measure, wake after sleep onset (WASO) could have been subtracted from time in bed. This was not assessed in the present study and is a limitation that could give an overestimation of the sleep duration. However, this is probably limited given that WASO is generally quite short during adolescence [22]. For insomnia, we relied on a broad range of questionnaire-based sleep parameters that were used in accordance with specific diagnostic definitions. A clinical interview is considered the gold standard and would have strengthened the validity of the insomnia category. The validity of the sleep measures could also have been strengthened by using a sleep diary with day-to-day functioning capturing nuances and variability regarding sleep outcomes. These limitations should be taken into consideration when interpreting the findings.

## Clinical implications and conclusion

Clinical implications relate to the question of whether we need to target sleep directly in the treatment of adolescents with anxiety and co-occurring sleep problems. The findings suggest that providers of early interventions should not assume that sleep problems in adolescents with elevated anxiety improve during anxiety-focused CBT. Therefore, sleep problems should be targeted more directly, and clinicians are advised to integrate sleep management strategies into the anxiety-focused CBT program. Attempts to integrate sleep management strategies and anxiety-focused CBT are rare but have promising results [32]. Thus, further evaluations are needed.

To conclude, no secondary effects on sleep outcomes in adolescents with anxiety were demonstrated during anxiety-focused GCBT for adolescents. Strategies that directly target sleep should be included in early intervention CBT for anxiety. Evaluation of the effects of CBT programs addressing both anxiety and co-occurring sleep problems in adolescents is warranted.

## Supplementary Information

Below is the link to the electronic supplementary material.

Supplementary file1

## Data Availability

The data may be available on reasonable request. To meet ethical requirements for the use of confidential patient data, all requests must be approved by the Norwegian Regional Committees for Medical and Health Research Ethics.
