# Supplementary material for: Effect of early intervention for anxiety on sleep outcomes in adolescents: a randomized trial
Source: Eur Child Adolesc Psychiatry. 2021 May 7;31(10):1–15. doi: 10.1007/s00787-021-01795-6 (PMC9532314; doi:10.1007/s00787-021-01795-6)
Supplement: Supplementary file 1 — Supplementary file1 [file 787_2021_1795_MOESM1_ESM.docx]

| Supplementary table S1. Studies on effects of anxiety focused cognitive behavioral therapy (CBT) on sleep problems in youth | | | | | | | |
| --- | --- | --- | --- | --- | --- | --- | --- |
| **Study** | **Country** | **Design** | **Participants** | **Sleep measure/ Informants** | **Intervention** | **Assess-ment** | **Findings** |
| Caporino et al., 2015 | USA | RCT comparing a) CBT b) Medication c) Medication & CBT d) Placebo | 488 Age 7- 17 yrs Mean:10,7 yrs Diagnoses GAD, SAD, SOP | Sleep composite score comprising: 1) Dysregulated Sleep 2) Separation related sleep problems (items from anxiety measures and CBCL). Parent (6 items) and youth (7 items) report | Individual CBT (Coping CAT), 14 sessions á 60 min for 12 weeks | Pre- and post | CBT reduced sleep related problems (small/medium effect sizes). Greatest reductions for combination therapy (c) reported by parents. Treatment responders for anxiety had greater reductions in sleep related problems. Changes in sleep differed by informant and type of problem. |
| Clementi et al, 2016 | USA | Open trial | 25 youth Age 7-16 yrs Mean: 9,9 yrs Anxiety symptoms or diagnoses SAD, GAD, SoP, SP, OCD, PD | 1) CSHQ - parent-report 2) PSAS-C - child-report | Early intervention, individual CBT (Acercamiento: Child and Adolescent Anxiety Program).12 sessions à 60 minutes. Mother and child present in sessions | Pre- and post | Significant change in global parent-reported sleep problems (e.g., bedtime resistance, sleep anxiety). Large effect size for total sleep problems, bedtime resistance, and sleep anxiety. Clinically meaningful change for 20%, with 45% above clinical cut-off at post-intervention. |
| Donovan et al., 2016 | Australia | RCT Online CBT vs WL | 63 children Age 7 to 12 yrs Mean: 9,5 yrs & 71 adolescents Age 12 - 18 yrs Mean:13,9 yrs Diagnoses SAD, SOP, GAD, SP | Sleep composite score comprising 7 items from CBCL. Parent report | Online individual CBT for anxiety (BRAVE) *Child*: 10 sessions + 5 parent session + 2 youth and 2 parent boosters *Adolescence*: 10 sessions + 5 parent sessions + 2 youth and 2 parent boosters | Pre-, post & 6 months follow-up | Children had significantly greater reduction in sleep problems from pre- to posttreatment compared to WL. Gains maintained at 6-month follow-up. For adolescents, parent-reported sleep problems reduced equally from pre- to post-assessment for treatment- and WL- group. |
| Ivarsson & Skarphedinsson, 2015 | Norway, Denmark, Sweden | Open trial | 269 youth Age: 7 to 17 yrs Diagnosis OCD | Sleep composite score comprising 6 items from CBCL and a sleep problem scale completed by parents | Individual CBT 14 weekly sessions á 75 min, including high level of parent participation | Pre- and post | Most sleep problems (e.g. nightmares, too little sleep, trouble sleeping) reduced during CBT for OCD |
| Kendall & Pimentel, 2003 | USA | Open trail | 47 youth Age 9 to 13 yrs Mean: 11.04 yrs Diagnoses GAD/OAD | A one-item measure of sleep disturbance from the ADIS-C/P symptoms checklist section of OAD/GAD | Individual CBT (Coping Cat), 16-20 sessions | Pre- and post | Decrease in somatic symptoms, including reduced sleep disturbance. |
| McMakin et al., 2019 | USA | Open trial | 133 (9-14 yrs, Mean: 10.96 yrs) Diagnoses GAD, SOP, SAD | CSHQ (parent report), SSR (child report), sleep diary (five nights), actigraphy | 16 session individual CBT (Coping Cat) or client-centered treatment (non-directive supportive therapy) | Pre-, mid- & post | Significant, but small effects, in sleep problems and improvements in subjective sleep patterns. Outcomes not clinically significant, and 75% of youth stayed above clinical cutoff. |
| Peterman et al, 2016 | USA | Open trial | 69 youth (completers only) Age: 7 to 17 yrs Mean: 10,9 yrs Diagnoses SAD, GAD, SoP, SP, OCD, PD, SM, AD-NOS | Parent report: CSHS and CSHQ Youth report: Sleep diary (2 weeks), ASHS, PSAS-C & SSR | Individual CBT (multiple manuals for anxiety depending on diagnosis; e.g., Coping Cat, Modular Approach to Therapy) 10 to 16 sessions dependent on treatment manual | Pre- and post | Improvement in parent-reported sleep problems (bedtime resistance). Responders to CBT for anxiety had greater improvement in sleep compared to non-responders. Youth reported lower sleep problems pre-treatment than parents, and no change pre- to post-treatment. |
| Storch et al., 2008 | USA | Open trial | 41 youth Age: 8 to 17 yrs Mean: 12,4 yrs Diagnosis OCD | Sleep composite score comprising 8 items from CBCL, MASC and CDI (both parent and child reports) | 14 sessions á 90-min CBT delivered weekly or intensive format. Parent(s) attending all sessions | Pre- and post | Total problems and most of the specific sleep problems (e.g., nightmares, has trouble sleeping) were reduced following cognitive-behavioral treatment |
| Note. AD-NOS= Anxiety Disorder-Not otherwise specified; C= child; CCT= Child Centered Therapy; CBT= Cognitive Behavioral Therapy; CBCL= | | | | | | | |
| Child Behavioral Checklist; CSHQ = Child’s Sleep Habits Questionnaire – Abbreviated Version; GAD= Generalized Anxiety Disorder; PSAS-C = | | | | | | | |
| Pre-Sleep Arousal Survey for Children; SM= Selective Mutism; SSR = Sleep Self-Report; CSHS= Children’s Sleep Hygiene Scale; ASHS= | | | | | | | |
| Adolescent Sleep Hygiene Scale; CCT= Child Centered Therapy; P= parent; SoP= Social Anxiety Disorder; SAD=Separation Anxiety Disorder; SP= | | | | | | | |
| Specific Phobia; OAD= Overanxious Disorder; OCD= Obsessive Compulsive Disorder; MASC= Multidimensional Anxiety Scale for Children; | | | | | | | |
| CDI= Children's Depression Inventory; PD=Panic Disorder; PSAS-C= Pre-Sleep Arousal Scale for Children; WL= wait list | | | | | | | |
